# ERβ1 represses basal-like breast cancer epithelial to mesenchymal transition by destabilizing EGFR

**DOI:** 10.1186/bcr3358

**Published:** 2012-11-16

**Authors:** Christoforos Thomas, Gayani Rajapaksa, Fotis Nikolos, Ruixin Hao, Anne Katchy, Catherine W McCollum, Maria Bondesson, Phil Quinlan, Alastair Thompson, Savitri Krishnamurthy, Francisco J Esteva, Jan-Åke Gustafsson

**Affiliations:** 1 Department of Biology and Biochemistry, Center for Nuclear Receptors and Cell Signaling, University of Houston, 3605 Cullen Blvd., Houston, TX 77204, USA; 2 Department of Surgery and Molecular Oncology, University of Dundee, DD1 9SY Dundee, UK; 3 Department of Pathology, The University of Texas MD Anderson Cancer Center, 1515 Holcombe Blvd., Houston, TX 77030, USA; 4 Department of Breast Medical Oncology, The University of Texas MD Anderson Cancer Center, 1515 Holcombe Blvd., Houston, TX 77030, USA

## Abstract

**Introduction:**

Epithelial to mesenchymal transition (EMT) is associated with the basal-like breast cancer phenotypes. Sixty percent of basal-like cancers have been shown to express wild-type estrogen receptor beta (ERβ1). However, it is still unclear whether the ERβ expression is related to EMT, invasion and metastasis in breast cancer. In the present study, we examined whether ERβ1 through regulating EMT can influence invasion and metastasis in basal-like cancers.

**Methods:**

Basal-like breast cancer cells (MDA-MB-231 and Hs578T), in which ERβ1 was either overexpressed or down-regulated were analyzed for their ability to migrate and invade (wound-healing assay, matrigel-coated Transwell assay) as well as for the expression of EMT markers and components of the EGFR pathway (immunoblotting, RT-PCR). Co-immunoprecipitation and ubiquitylation assays were employed to examine whether ERβ1 alters epidermal growth factor receptor (EGFR) protein degradation and the interaction between EGFR and the ubiquitin ligase c-Cbl. The metastatic potential of the ERβ1-expressing MDA-MB-231 cells was evaluated *in vivo *in a zebrafish xenotransplantation model and the correlation between ERβ1 and E-cadherin expression was examined in 208 clinical breast cancer specimens by immunohistochemistry.

**Results:**

Here we show that ERβ1 inhibits EMT and invasion in basal-like breast cancer cells when they grow either *in vitro *or *in vivo *in zebrafish. The inhibition of EMT correlates with an ERβ1-mediated up-regulation of miR-200a/b/429 and the subsequent repression of ZEB1 and SIP1, which results in increased expression of E-cadherin. The positive correlation of ERβ1 and E-cadherin expression was additionally observed in breast tumor samples. Down-regulation of the basal marker EGFR through stabilization of the ubiquitin ligase c-Cbl complexes and subsequent ubiquitylation and degradation of the activated receptor is involved in the ERβ1-mediated repression of EMT and induction of EGFR signaling abolished the ability of ERβ1 to sustain the epithelial phenotype.

**Conclusions:**

Taken together, the results of our study strengthen the association of ERβ1 with the regulation of EMT and propose the receptor as a potential crucial marker in predicting metastasis in breast cancer.

## Introduction

In the last decade, genomic studies have identified five breast cancer intrinsic subtypes (Luminal A, Luminal B, HER2 (overexpressing the *ERBB2*), basal-like and claudin-low) [1,2]. In a recent study, an integrated analysis of copy number and gene expression split the intrinsic subtypes revealing novel subgroups with distinct clinical outcome, including a high-risk ERα-positive subgroup and a subset of ERα-positive and ERα-negative cases with a favorable outcome. According to this analysis, the majority of the basal-like tumors formed a high-genomic instability subgroup with relatively good long-term outcomes (after five years) [3]. Basal-like phenotypes represent tumors that express markers that are characteristic of the myoepithelium of the normal mammary gland, such as epidermal growth factor receptor (EGFR), p63 and the basal cytokeratins CK14, CK5/6 and CK17 [1,4]. They show partial overlap with the triple-negative breast cancers that are characterized by a lack of HER2 gene amplification and estrogen and progesterone receptor expression. Approximately 75% of triple-negative breast cancers are classified as basal-like tumors on the basis of their overall gene-expression profile. The basal-like phenotype represents a more homogeneous group of cancers than the group of cancers defined by triple negativity [5]. Basal-like tumors are often resistant to chemotherapy and develop distant metastases in characteristic tissues, such as lung and brain [6]. Recent studies have suggested a correlation between the basal phenotypes and epithelial to mesenchymal transition (EMT) [7].

EMT has been reported to promote invasion during the progression of breast carcinomas and it is considered as an essential early step in tumor metastasis [8,9]. EMT is characterized by loss of cellular adhesion, which is mediated by down-regulation of adhesion molecules, such as CD44 and E-cadherin [10,11]. The expression of E-cadherin is regulated by a number of transcriptional repressors, which include SNAIL, SLUG, SIP-1 (ZEB-2), δEF1 (ZEB-1) and TWIST [12-15]. The family of microRNAs 200 (miR-200a, miR-200b, miR-200c, miR-141 and miR-429) and the miR-205A regulate the expression of the transcriptional repressors of E-cadherin ZEB-1 and ZEB-2 and, consequently, the levels of E-cadherin in breast cancer cells and tissues. A decrease in the expression of these microRNAs has been observed in cells that have undergone EMT and in mesenchymal regions of metaplastic breast cancer lacking E-cadherin expression [16]. Up-regulation of components of the EGFR signaling pathway, such as ERK2, has also been reported to influence the levels of E-cadherin by regulating the transcriptional repressors ZEB-1 and ZEB-2 [17,18].

The potential role of estrogen receptors in regulating EMT and aggressive behavior in breast cancer has recently been under investigation [19]. Although a decline of ERα levels is detected in invasive breast cancers, a few studies have shown regulation of cell migration and invasion by ERα [20,21]. Recent studies have also associated the ERβ isoforms ERβ1, ERβ2 and ERβ5 with the regulation of cell migration and invasion in prostate cancer [22,23]. Down-regulation of the fully functional ERβ isoform ERβ1 (also known as wild-type ERβ) promoted EMT in prostate cancer cells and this correlated with the loss of ERβ1 in high Gleason grade invasive prostate carcinoma [22]. Interestingly, patients with triple-negative breast cancer that were treated with adjuvant tamoxifen have been shown to have significantly better survival when the tumors were positive for ERβ1 [24]. In addition, clinical findings showed an inverse correlation between ERβ1 positivity and expression of EGFR, a crucial component in basal-like cancers that drives proliferation and EMT [25]. Given that down-regulation of ERβ1 has been observed in invasive breast cancers, in this study we hypothesized that ERβ1 functions to maintain an epithelial phenotype in breast cancer and examined whether ERβ1 reduces the invasiveness of basal cancer cells by repressing EMT [26].

## Materials and methods

### Cells, reagents and transfections

The breast cancer cell lines (MDA-MB-231, Hs578T and MCF-7) and the lung cancer cell line (H1299) were obtained from the ATCC. In 17β-estradiol (E2) experiments, cells were maintained in phenol red-free media containing two or five percent dextran-coated charcoal (DCC)-treated fetal calf serum (FCS). Transforming growth factor β (TGF-β) and EGF experiments were performed in serum-free or 0.5% FCS media with recombinant human TGF-β1 (5 ng/ml; R & D Systems, Minneapolis, MN USA) for one to three days or EGF (10 ng/ml; Sigma) for 24 h. MDA-MB-231 and Hs578T cells were infected with lentiviruses containing the plenti6/V5 empty vector or the recombinant pLenti6/V5-D-FLAG-ERβ1 and pLenti6/V5-D-FLAG-ERα plasmids as described previously [27]. Cells were transfected twice with ERβ-specific siRNAs (Invitrogen, Carlsbad, CA USA), target sequences 1# 5'-TTAGCGACGTCTGTCGCGTCTTCAC-3'; 2# 5'-TTACGACATTAAGTAGTGTCGTCCC-3'; 3# 5'-TATTGACCGCTACCTGGTGATTTCC-3'. siRNA targeting luciferase was used as a control (Cat. No. 12935-146, Invitrogen). For the expression of wild-type EGFR, cells were stably transfected with the pBABE-EGFR construct (Addgene, plasmid # 11011, Cambridge, MA USA), using the empty pBABE vector (Addgene, plasmid # 1764) as a control. Cells were transfected with microRNA inhibitors at a final concentration of 300 nM (100 nM of each of miR-200a, miR-200b and miR-429 2'-O-Methylmodified oligonucleotides, Dharmacon, Waltham, MA USA) or a negative control inhibitor (300 nM). The complementary sequences for miR-200a, miR-200b and miR-429 were cloned in the 3' end of the luciferace gene into the PGL3-promoter vector (Promega, San Luis Obispo, CA USA). A total of 2 × 10^5 ^cells were seeded at 24-well plates and transfected with 800 ng DNA/well (PGL3, β-gal) as well as microRNA inhibitors. Twenty-four hours after transfection, cells were lysed and analyzed using a Luciferase Assay (Promega). Luciferase units were normalized to β-galactosidase units. For ERE-luciferase reporter assays, cells were incubated in DCC-FCS media for 48 h and transfected with 800 ng DNA/well (3-ERE-TATA-LUC reporter plasmid, β-gal plasmid) using LipofectamineTM 2000 (Invitrogen). Cells were mock treated (EtOH) or treated with E2 for 24 h in 2% DCC-FCS media. Reporter gene activity was normalized to β-galactosidase enzyme activity.

### Migration and invasion assays

In the wound-healing assay, cells were allowed to form monolayers at 24-well plates. The monolayer was scratched with a pipette tip to form the wound. Twelve hours later, images of the wound were taken using a 10× objective in an OLYMPUS IX51 microscope equipped with an OLYMPUS camera (OLYMPUS, Center Valley, PA USA) and cells in the wound area in five independent fields were counted.

In the invasion assay, cells were seeded in matrigel-coated 6.5 mm Transwell champers (8 μm pore size; BD Biosciences, San Jose, CA USA). Six hours later, the cells that had been translocated to lower compartments of the wells and attached to the lower surface of the filter were fixed in methanol and stained with crystal-violet. The stained cells were counted in five independent fields in each Transwell.

### Immunofluorescence and microscopy

Cells were plated onto 18 mm^2 ^coverslips, fixed in 3% paraformaldehyde (PFA) and 2% sucrose for 15 minutes at room temperature (RT), permeabilized in 20 mM Tris HCI pH 7.5, 75 mM NaCl, 300 mM sucrose, 3 mM MgCl_2 _and 0.5% Triton-X-100 for 15 minutes at RT and blocked with 5% goat serum in phosphate-buffered saline (PBS) for 1 h at RT. Slides were stained with an E-cadherin antibody (BD Biosciences) at 4°C overnight, washed, incubated with secondary antibody and images were collected on an OLYMPUS BX51 microscope equipped with an OLYMPUS XM10 camera (OLYMPUS, Center Valley, PA USA).

### RNA extraction and real-time PCR

Total RNA was isolated using TriZol reagent (Invitrogen) and reverse-transcribed to cDNA using a SuperScript™ II reverse transcriptase kit (Invitrogen). Real-Time PCR was performed using the SyBr green PCR kit (Applied Biosystems, Grand Island, NY USA). EGFR mRNA levels were additionally analyzed using TaqMan mRNA assay according to the manufacturer's instructions (Applied Biosystems). All quantitative data were normalized to GAPDH and actin-β. For microRNAs, real-time PCR was performed as above using TaqMan microRNA assays (Applied Biosystems). All microRNA data are expressed relative to a U6 small nuclear (sn) RNA TaqMan PCR performed on the same sample. The sequences of the primers used for qPCR are listed in the Additional file 1, Table S1.

### Immunoblotting and immunoprecipitation

Cells were lysed in RIPA buffer, including protease and phosphatase inhibitors as previously described [28]. For separation of cytoplasmic and nuclear fractions, cells were suspended in a cold buffer containing 10 mM Hepes pH 7.0, 10 mM KCI, 0.1 mM EDTA, 1 mM DTT and 0.5 mM PMSF. After 15 minutes' incubation on ice, the homogenate was mixed with 10% NP-40 and centrifuged for 30 sec. The nuclear pellet was resuspended in a cold buffer containing 10 mM Hepes-KOH pH 7.9, 400 mM NaCI, 0.1 mM EDTA, 5% glycerol, 1 mM DTT and 0.5 mM PMSF and the nuclear extract was isolated by centrifugation. The blots were performed as previously described [28]. Primary antibodies used in immunoblotting include: ERα, E-cadherin, N-cadherin, cadherin-11, vimentin, ZEB-1, SIP1, Lamin A/C and Tubulin (Santa Cruz Biotechnology, Santa Cruz, CA USA), actin-β (Sigma St. Louis, MO USA), EGFR (Santa Cruz Biotechnology, Cell Signaling, Danvers, MA USA), ERβ1 (14C8; GeneTex, Irvine, CA USA), which detects an N-terminal epitope and recognizes the ERβ isoforms derived from alternative splicing of the last exon, including ERβ1 and an in-house antibody that detects an epitope in ligand binding domain of ERβ1 (amino acids 320 to 527)) [29], SNAIL (Abcam, Cambridge, MA USA), p-ERK1/2 and total ERK1/2 (Cell Signaling), c-Cbl (BD Biosciences). Recombinant ERβ1 (Invitrogen) was loaded in SDS-PAGE gels and used as a positive control. For ubiquitylation analysis, cells were lysed in RIPA buffer containing protease inhibitor cocktail (Roche, Branchburg, NJ USA). The lysates were briefly sonicated and cleared by centrifugation at 4°C. Supernatants were incubated with anti-EGFR antibody overnight at 4°C and A/G agarose beads for 2 h at 4°C. The immunocomplexes were washed three times, boiled in 2× sample buffer and immunoblotted with anti-ubiquitin antibody (Santa Cruz Biotechnology). For the EGFR-c-Cbl co-immunoprecipitations, cells were lysed in a buffer containing 50 mM Hepes pH 7.4, 150 mM NaCI, 1 mM EDTA, 1 mM EGTA, 1% Nonidet P-40, 1% glycerol including protease and phosphatase inhibitors. Lysates were incubated on ice for 30 minutes without sonication, cleared by centrifugation and the cleared lysates were subjected to immunoprecipitation as described.

### Zebrafish tumor model and generation of fluorescent cells

Animal work was approved by the Institutional Animal Care and Use Committee (IACUC) at the University of Houston. Control (Lenti) and ERβ1-expressing (ERβ1) MDA-MB-231 cells were stably transfected with either the pAmCyan vector or the pCMCV-DsRed vector (Clontech, Mountain View, CA USA). A tumor cell suspension (5 nL) of approximately 300 to 500 cells containing a mixture of equal numbers of either DsRed-Lenti:AmCyan-ERβ1 cells or AmCyan-Lenti:DsRed-ERβ1 cells were injected into the perivitelline cavity of each 48 h post-fertilization casper *Tg(Flk-1;EGFP) *anesthetized embryo using a pressure injector (Harvard Apparatus, Holliston, MA USA) and Manipulator (MM33-Right, Märzhäuser Wetzlar, Wetzlar, Germany). Glass needles (1.00 mm in diameter, Sutter Instrument Company, Novato, CA USA), were used for the microinjection. Injected embryos were kept at 32°C and were examined every day for tumor invasion using a fluorescent microscope (OLYMPUS IX51) equipped with an OLYMPUS XM10 camera. Information for the zebrafish lines is included in the Additional file 2, Supplementary materials and methods.

### Patient information

A tissue microarray consisting of 240 breast cancer samples was constructed by the Tayside Tissue Bank. Access to tumor samples was approved by the Tayside Regional Ethics Committee with written informed consent from contributing patients. Clinical history and tumor characteristics were available for 238 cases. The clinicopathological characteristics of these patients are summarized in the Additional file 3, Table S2. The majority of the patients received adjuvant endocrine therapy or combined endocrine therapy and chemotherapy, with or without radiotherapy. Among these patients, 74.7% were ERα-positive, 53.7% were PR-positive and 14.5% were HER2-positive. Histologically, 192 invasive ductal carcinomas (80.6%), 14 invasive lobular carcinomas (5.8%), 5 tubular carcinomas (2.1%), 5 mucinous carcinomas (2.1%) and 22 other histological or mixed types (9.2%) were included.

### Antibody validation and immunohistochemistry

The anti-ERβ1 antibody (clone PPG5/10, Dako, Carpinteria, CA USA), which is specific for the C-terminal amino acid sequences of ERβ1, was used for immunohistochemistry (IHC). This antibody was validated by immunocytochemistry. Briefly, H1299 human lung cancer cells were stably transfected with the pIRES empty vector (Clontech) or the recombinant pIRES-ERβ1 or pIRES-ERβ2 plasmids. Control, ERβ1 and ERβ2-expressing cells were fixed with 10% formalin. The cell suspension was centrifuged and the cell pellet was folded in sharkskin filter paper using four overlapping edges and placed within the base of a tissue cassette. The cassette was placed in a specimen bucket with 10% formalin. The formalin-fixed cell material was embedded in paraffin, cut at 5 μm intervals and used for H&E staining and IHC.

For immunohistochemistry, formalin-fixed, paraffin-embedded sections were de-paraffinized with xylene and rehydrated through a graded alcohol series. For antigen retrieval, the slides were immersed in 10 mM sodium citrate buffer (pH 6.0) and maintained at a sub-boiling temperature for six minutes. The endogenous peroxidase activity was blocked by incubation in 0.3% hydrogen peroxide solution for 20 minutes. The slides were first incubated with 1% bovine serum albumin (BSA) to block non-specific staining and then with the primary antibody overnight at 4°C in a humidified chamber. The sections were then processed according to the Dako DAB detection kit.

The results of the immunohistochemistry were assessed by a pathologist (SK) in a blinded fashion. Each specimen was assigned a score according to the intensity of the nuclear staining (for ERβ1) and cytoplasmic and membrane staining (for E-cadherin) (no staining = 0, weak staining = 1, moderate staining = 2, strong staining = 3) and the extent of stained cells (0% = 0, 1 to 24% = 1, 25 to 49% = 2, 50 to 74% = 3, 75 to 100% = 4). The final immunoreactive score was determined by multiplying the intensity score with the extent of the score of stained cells, ranging from 0 (the minimum score) to 12 (the maximum score). We defined ERβ1 expression as low (score 0 to 4), medium (score 5 to 8) and high (score 9 to 12). For E-cadherin, we defined a 0 score as negative and a 1 to 12 as positive.

### Statistical analysis

The correlation between expression of ERβ1 and E-cadherin, respectively, was determined using Pearson's correlation test. All statistical tests were two-sided and *P-*values less than or equal to 0.05 were considered as statistically significant. The statistical analyses were performed using SPSS 20.0 software (SPSS, IBM, Armonk, NY USA).

## Results

### ERβ1 is required for the epithelial breast cancer phenotype

**Figure 1:**
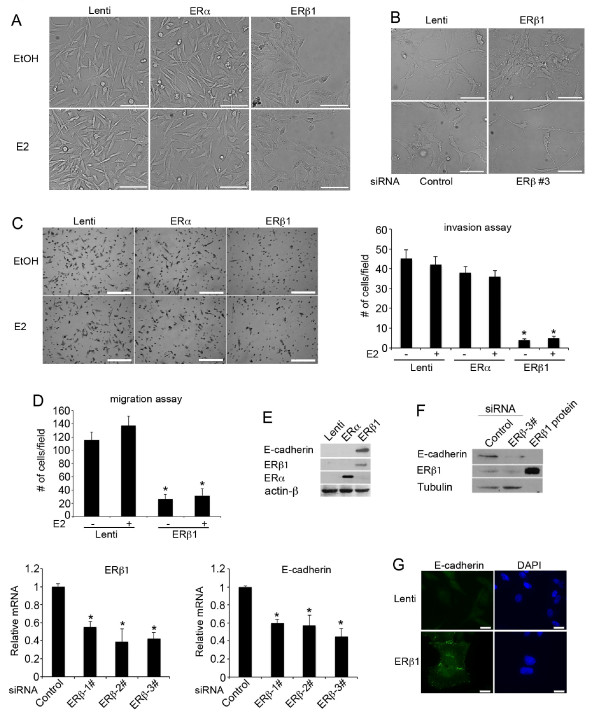
**ERβ1 inhibits invasion and migration in breast cancer cells by regulating EMT**. **(A) **Control (Lenti), ERα- and ERβ1-expressing MDA-MB-231 cells following incubation with EtOH or 17β-estradiol (E2) for 24 h (scale bars, 50 μm). **(B) **Control (Lenti) and ERβ1-expressing Hs578T cells (upper panel) and Hs578T cells that were transiently transfected with a siRNA targeting luciferase (Control) or a specific ERβ siRNA (siRNA 3#) (lower panel) were photographed (scale bars, 100 μM). **(C) **Control (Lenti), ERα- and ERβ1-expressing MDA-MB-231 cells were incubated with EtOH or E2 and assessed for invasion by using matrigel-coated Transwell chambers. The cells that were translocated to the lower surface of the filter were shown (left panel) (scale bars, 500 μm). The graph shows the mean (cell number per field) of three separate experiments with the standard error of the mean (SEM) and *P-*value (*) ≤0.05% indicated. **(D) **Control (Lenti) and ERβ1-expressing MDA-MB-231 cells were incubated with E2 for 24 h and assessed for migration using wound-healing assay. The bar graph shows the mean (cells migrated into the wound) of three separate experiments with SEM and *P-*value (*) ≤0.05% indicated. **(E) **E-cadherin protein levels in control (Lenti), ERα- or ERβ1-expressing MDA-MB-231 cells. **(F) **E-cadherin expression was analyzed by immunoblotting in MDA-MB-231 cells transfected with control or ERβ siRNA (3#) (upper panel) and qPCR in MDA-MB-231 cells transfected with control or three specific ERβ siRNAs (lower panel). The graph indicates the mean of three separate experiments with SEM and *P-*value (*) ≤0.05%. **(G) **E-cadherin was visualized by immunofluorescence in control (Lenti) and ERβ1-expressing cells (scale bars, 20 μm).

**Figure 2:**
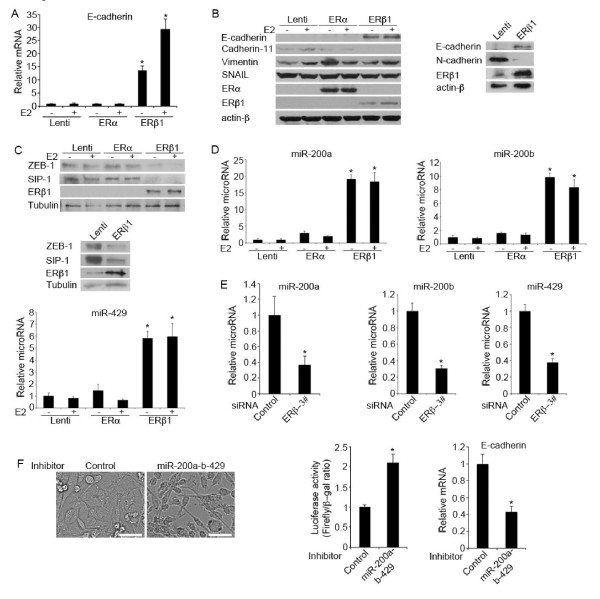
**ERβ1 induces the expression of E-cadherin by up-regulating members of the microRNA 200 family and repressing the expression of ZEB-1 and SIP-1**. **(A) **E-cadherin mRNA levels in control (Lenti), ERα- and ERβ1-expressing MDA-MB-231 cells following incubation with or without E2 for 24 h. The graph shows the mean of three separate experiments with SEM and *P-*value (*) ≤0.05% indicated. **(B) **Left panel: protein levels of EMT markers in control (Lenti), ERα- and ERβ1-expressing MDA-MB-231 cells following incubation with or without E2 for 24 h. Right panel: E- and N-cadherin protein levels in control (Lenti) and ERβ1-expressing Hs578T cells. **(C) **ZEB-1 and SIP-1 protein levels in control (Lenti), ERα- and ERβ1-expressing MDA-MB-231 cells (upper panel) and in control and ERβ1-expressing Hs578T cells (lower panel). **(D) **Control, ERα- and ERβ1-expressing MDA-MB-231 cells were analyzed for miR-200a, miR-200b and miR-429 expression by qPCR. The graphs show data as fold change compared with the untreated Lenti cells (mean of three separate experiments with SEM and *P-*value (*) ≤0.05% indicated). **(E) **MDA-MB-231 cells were transiently transfected with control or ERβ siRNA (3#) and analyzed for miR-200a, miR-200b and miR-429 expression by qPCR. The graphs show the mean of three separate experiments with SEM and *P-*value (*) ≤0.05% indicated. **(F) **ERβ1-expressing MDA-MB-231 cells were transfected with inhibitors of miR-200a, miR-200b and miR-429 or a negative control inhibitor. Cells were photographed and analyzed for E-cadherin expression by qPCR (scale bars, 50 μm). The level of functional knockdown of miR-200a-b-429 was examined by a miR-200a-b-429-regulated reporter assay. The graphs show the mean of three separate experiments with SEM and *P-*value (*) ≤0.05% indicated.

Basal-like phenotypes are high-grade (grade III), ERα-negative invasive breast tumors that express EMT markers and show cadherin switching as a consequence of tumor de-differentiation [7]. Previous studies have shown a decline of ERβ1 expression from ductal carcinoma *in situ *(DCIS) to invasive cancer and an association of the receptor with the repression of mesenchymal characteristics in invasive prostate cancer [22,30,31]. We hypothesized that ERβ1 regulates EMT in breast cancer and that low ERβ1 expression in a proportion of basal-like cancers is associated with mesenchymal characteristics and poor clinical outcome. To test this hypothesis, we stably expressed ERβ1 in the invasive triple-negative breast cancer MDA-MB-231 and Hs578T cells and compared the expression levels achieved in these cells with the endogenous expression of ERs in MCF-7 cells (Additional file 4, Figure S1). According to recent studies, MDA-MB-231 and Hs578T cells most resemble the claudin-low breast cancer subtype; however, as basal-like tumors, they display low expression of the luminal and HER2 gene clusters and express low amounts of ERβ1 [32]. Induction of ERβ1 expression promoted morphological changes in these cells characterized by the loss of the "fibroblastoid-like" phenotype and the acquisition of an epithelial-like compact morphology (Figure 1A, B, upper panel). Furthermore, a more spindle-shaped morphology was observed when endogenous ERβ1 was knocked down with ERβ siRNA in Hs578T cells (Figure 1B, lower panel). Induction of ERβ1 expression altered the morphology of the MDA-MB-231 and Hs578T cells in the absence of ligand. The morphology of the ERβ1-expressing MDA-MB-231 cells following treatment with 17β-estradiol (E2) was similar to that of the untreated cells (Figure 1A). Consistent with the changes in the morphology, induction of ERβ1 expression in MDA-MB-231 cells repressed invasion and migration (Figure 1C, D), functions characteristic of EMT [33]. Although induction of ERβ1 and ERα expression resulted in a similar activation of an ERE-luciferase reporter, ERα failed to promote epithelial morphology and reduce the invasiveness of MDA-MB-231 cells (Figure 1A, C; Additional file 4, Figure S1; Additional file 5, Supplementary figure legends). Similar to the impact on the cellular morphology and invasiveness, only ERβ1 inhibited cadherin switching as shown by the up-regulation of epithelial E-cadherin in both MDA-MB-231 and Hs578T cells and down-regulation of the mesenchymal cadherin-11 in MDA-MB-231 and N-cadherin in Hs578T cells (Figures 1E and 2A, B; Additional file 6 Figure S2A). The positive correlation between ERβ1 and E-cadherin expression was confirmed by the decrease of E-cadherin mRNA and protein levels when ERβ1 was knocked down in MDA-MB-231 cells (Figure 1F). In line with the results from the immunoblotting analysis, immunofluorescence showed higher expression of E-cadherin in the cell surface of the ERβ1-expressing cells compared to the control cells (Figure 1G). This suggests that ERβ1 up-regulates the functional form of E-cadherin that promotes cell-cell adhesion. No alteration in the levels of the mesenchymal marker vimentin was detected in ERβ1-expressing MDA-MB-231 cells suggesting that ERβ1 induces cell-cell adhesion in these cells by primarily regulating the expression of cadherin (Figure 2B; Additional file 6 Figure S2B).

### miR-200 and ZEB1/2 are involved in ERβ1-mediated regulation of E-cadherin

A number of transcription factors (SNAIL, SLUG, TWIST, SIP-1 and ZEB-1) have been shown to promote EMT *in vitro *by acting as transcriptional repressors of E-cadherin [34,35]. Nuclear translocation of SNAIL has been shown to repress E-cadherin expression in ERβ1 knockdown prostate cancer cells [22]. Based on these data, we examined whether SNAIL inhibition is involved in the ERβ1-mediated induction of E-cadherin expression that we observed in breast cancer cells. Surprisingly, induction of ERβ1 expression in MDA-MB-231 cells neither altered the expression nor the intracellular localization of SNAIL as assessed by immunoblotting using cytoplasmic and nuclear extracts from control and ERβ1-expressing cells as well as immunofluorescence microscopy using a SNAIL Ab (Additional file 7, Figure S3A, B). Instead, up-regulation of ERβ1 in MDA-MB-231 and Hs578T cells repressed the expression of the transcriptional repressors of E-cadherin ZEB-1 and SIP-1 (Figure 2C). Given that recent studies have reported that the microRNA-200 family and miR-205 regulate EMT by targeting ZEB-1 and SIP-1, we examined whether the expression of members of the microRNA-200 family and miR-205 were up-regulated prior to repression of ZEB-1 and SIP-1 expression in ERβ1-expressing cells [16]. Using quantitative real-time PCR we found that the cluster of miR-200b-200a-429 was up-regulated by more than 7-fold in the ERβ1-expressing MDA-MB-231 and Hs578T cells (3.5-fold increase only for miR-429 in Hs578T cells) (Figure 2D; Additional file 8, Figure S4A). In addition, reduction of endogenous ERβ1 expression in MDA-MB-231 and Hs578T cells by ERβ siRNA led to a decrease in the expression of miR-200a, miR-200b and miR-429 (Figure 2E; Additional file 8, Figure S4B). In contrast to the cluster of miR-200b-200a-429, the cluster miR-200c-141 and the miR-205 were unchanged in ERβ1-expressing MDA-MB-231 cells (Additional file 9, Figure S5). We also examined how important is the up-regulation of miR-200a-b-429 for the ERβ1-mediated repression of EMT. We transfected the ERβ1-expressing MDA-MB-231 cells with inhibitors of miR-200a, miR-200b and miR-429 and assessed the level of functional knockdown of miR200a-b-429 by a reporter assay, in which the complementary sequence of miR200a-b-429 was introduced in the 3' UTR of a luciferase reporter gene. Transfection of the cells with miR200a-b-429 inhibitors resulted in a more than two-fold increase in luciferase activity compared with the negative control inhibitor suggesting that a greater than 50% inhibition of the miR200a-b-429 function had been achieved by the miR200a-b-429 inhibitors (Figure 2F). Inhibition of miR200a-b-429 partially reversed the ERβ1-mediated epithelial phenotype and caused a 50% reduction in the expression of E-cadherin (Figure 2F). These data strengthen the role of ERβ1 in regulating EMT and suggest a mechanism through which the receptor may regulate E-cadherin expression.

### ERβ1 inhibits EMT by repressing EGFR signaling

**Figure 3:**
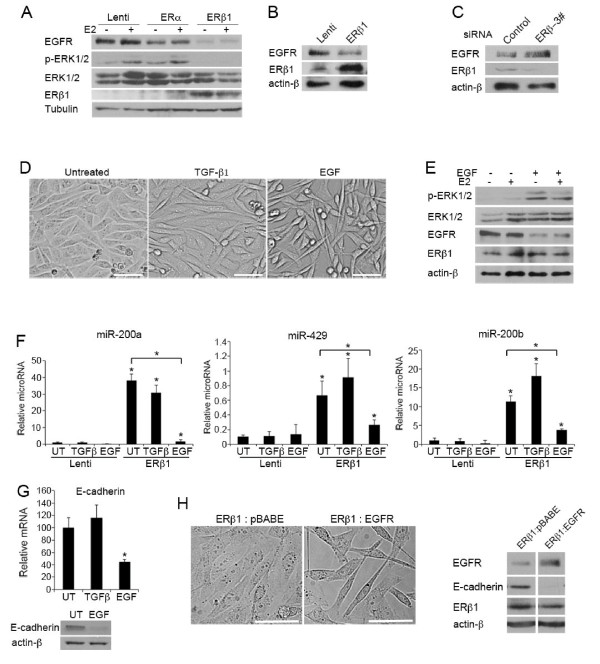
**EGFR promotes EMT and its down-regulation is involved in ERβ1-induced E-cadherin expression**. **(A) **EGFR, total ERK1/2 and phospho-ERK1/2 levels in control (Lenti), ERα- and ERβ1-expressing MDA-MB-231 cells following incubation with or without E2 for 24 h. **(B) **EGFR protein levels in control (Lenti) and ERβ1-expressing Hs578T cells. **(C) **EGFR protein levels in MDA-MB-231 cells transiently transfected with control or ERβ siRNA (3#). **(D) **ERβ1-expressing MDA-MB-231 cells were incubated in absence or presence of 5 ng/ml TGF-β1 or 10 ng/ml EGF for 24 h and photographed (scale bars, 50 μm). **(E) **ERβ1-expressing MDA-MB-231 cells were incubated in absence or presence of 10 ng/ml EGF for 24 h and analyzed for the expression of EGFR, total ERK1/2 and phospho-ERK1/2 by immunoblotting. Note that the decreased EGFR levels following EGF treatment is due to increased degradation. **(F) **miR-200a, miR-200b and miR-429 levels in control (Lenti) and ERβ1-expressing MDA-MB-231 cells following incubation with 5 ng/ml TGF-β1 or 10 ng/ml EGF for 24 h. The graph shows the data as fold change compared with the untreated Lenti cells (mean of three separate experiments (± SEM) with *P-*value (*) ≤0.05%). **(G) **E-cadherin mRNA and protein levels in ERβ1-expressing MDA-MB-231 cells following incubation with 5 ng/ml TGF-β1 or 10 ng/ml EGF for 24 h. The graph shows the mean of three separate experiments with SEM and *P-*value (*) ≤0.05% indicated. **(H) **ERβ1-expressing MDA-MB-231 cells were stably co-transfected with an empty pBABE vector (ERβ1:pBABE cells) or the pBABE-EGFR plasmid (ERβ1:EGFR cells), photographed and analyzed for EGFR, E-cadherin and ERβ1 expression by immunoblotting (scale bars, 50 μm).

EGFR that is overexpressed in MDA-MB-231 and Hs578T cells has been associated with poor survival in basal-like breast cancers (Additional file 10, Figure S6). Overexpression of EGFR is known to promote migration in breast cancer cells [36,37]. Activation of EGFR following ligand binding results in phosphorylation and activation of extracellular-signal-regulated kinases (ERKs) [38]. Activation of ERK2 has recently been shown to promote EMT by inducing the expression of the transcriptional repressors of E-cadherin ZEB-1 and SIP-1 [17,18]. Given the repression of ZEB-1 and SIP-1 expression observed in ERβ1-expressing MDA-MB-231 and Hs578T cells, we examined whether ERβ1 inhibits EMT by down-regulating EGFR signaling. Induction of ERβ1 expression caused a strong reduction in the EGFR protein levels in MDA-MB-231 and Hs578T cells and decreased the phosphorylation of ERK1/2 as assessed by immunoblotting using an ERK1/2 phospho-specific antibody (Figure 3A, B). Furthermore, reduction of endogenous ERβ1 expression in MDA-MB-231 cells led to up-regulation of EGFR (Figure 3C). Analysis of EGFR mRNA by qPCR showed the same levels in control and ERβ1-expressing cells as well as in cells where ERβ1 had been knocked down, suggesting that ERβ1 does not regulate the transcription of *EGFR *gene (Additional file 6, Figure S2C). To test whether the ERβ1-EGFR interaction is a critical regulator of EMT in basal-like breast cancer cells, we treated the ERβ1-expressing cells with EGF or the EMT inducer TGF-β1 for 24 h. For the same purpose, we stably transfected the ERβ1-expressing MDA-MB-231 cells with an empty vector or a plasmid that encodes wild-type EGFR. As expected, treatment of the cells with EGF restored the phosphorylation of ERK1/2, decreased the cell-cell contact observed in the ERβ1-expressing cells and abolished the ERβ1-mediated up-regulation of miR-200a-200b-429 and the increased levels of E-cadherin (Figure 3D-F, 3G). In contrast, treatment of the cells with TGF-β1, for the same time period as for EGF, failed to reverse the ERβ1-induced phenotype in MDA-MB-231 cells (Figure 3D, F, G). As in the case of EGF treatment, EGFR overexpression induced a more fibroblastoid morphology in ERβ1-expressing cells, which was accompanied by down-regulation of E-cadherin (Figure 3H).

### ERβ1 induces degradation of EGFR by enhancing the EGFR-c-Cbl interaction

**Figure 4:**
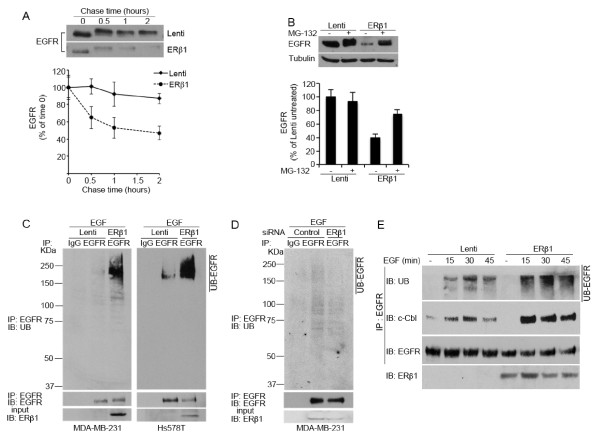
**ERβ1 induces ubiquitylation and degradation of EGFR**. **(A) **Control (Lenti) and ERβ1-expressing MDA-MB-231 cells were incubated in the presence of 100 μM cycloheximide and 10 ng/ml EGF for the indicated times and analyzed for EGFR expression by immunoblotting. Treatment with EGF induces phosphorylation of EGFR and this accounts for the retarded electrophoretic mobility of EGFR at times 0.5 to 2. Lower panel: the graph represents the quantification of EGFR protein abundance from three independent experiments with SEM and *P-*value (*) ≤0.05% indicated. **(B) **Control (Lenti) and ERβ1-expressing MDA-MB-231 cells were incubated in absence or presence of 1 μM MG-132 for 6 h and analyzed for EGFR expression by immunoblotting. Lower panel: the bar graph represents the quantification of EGFR protein levels with SEM and *P-*value (*) ≤0.05% indicated. **(C) **Control (Lenti) and ERβ1-expressing MDA-MB-231 and Hs578T cells were incubated in the presence of 10 ng/ml EGF for 20 minutes. Lysates were immunoprecipitated with anti-EGFR antibody, followed by immunoblotting with the indicated antibodies. The bottom panel is the input control of cell lysates. **(D) **MDA-MB-231 cells were transiently transfected with control or ERβ siRNA (3#). 72 h after the transfection, cells were incubated with 10 ng/ml EGF for 20 minutes and analyzed as described in C. **(E) **Control (Lenti) and ERβ1-expressing MDA-MB-231 cells were serum starved, challenged with 10 ng/ml EGF for the indicated times and lysed under nondenaturing conditions. EGFR immunoprecipitates were probed with antibodies against EGFR and c-Cbl. The bottom panel is the input control of cell lysates.

Given that ERβ1 altered only the protein but not the mRNA levels of EGFR, we set out to investigate whether ERβ1 regulates EGFR at a post-transcriptional level. Specifically, we hypothesized that ERβ1 induces degradation of the EGFR protein. EGFR degradation occurs through a process that includes ubiquitylation of the receptor, accelerated endocytosis and degradation by proteasomal and lysosomal hydrolases [39]. In chase experiments, expression of ERβ1 reduced the half-life of EGFR suggesting that EGFR protein turnover was enhanced by ERβ1 (Figure 4A). Treatment of the cells with the proteasome inhibitor MG-132 inhibited the ERβ1-dependent reduction in EGFR protein abundance (Figure 4B) confirming that EGFR down-regulation in ERβ1-expressing cells was due to increased degradation. Given that ubiquitylation is an important step in the degradation of EGFR, we carried out ubiquitylation assays to test whether ERβ1 induces ubiquitylation of EGFR. Interestingly, the levels of the ubiquitylated EGFR were dramatically increased in ERβ1-expressing MDA-MB-231 and Hs578T cells (Figure 4C). Furthermore, the ubiquitylated EGFR was decreased when ERβ1 was knocked down in MDA-MB-231 cells (Figure 4D). Given that ubiquitylation of the activated EGFR is mediated by members of the Cbl family of RING domain E3 ubiquitin ligases, including the c-Cbl [40], we examined whether ERβ1 promotes ubiquitylation of EGFR by inducing its association with c-Cbl. In control MDA-MB-231 cells, immunoprecipitation of EGFR under nondenaturing conditions showed a rapid but transient recruitment of c-Cbl to EGFR with a barely detectable c-Cbl-EGFR association at 45 minutes following EGF induction. ERβ1-expressing MDA-MB-231 cells showed enhanced and more sustained c-Cbl-EGFR association with high amounts of c-Cbl recruited to EGFR even at 45 minutes following EGF induction (Figure 4E). These results strengthen our hypothesis that ERβ1 down-regulates EGFR by inducing its degradation.

### ERβ1 inhibits invasion of breast cancer cells *in vivo *

**Figure 5:**
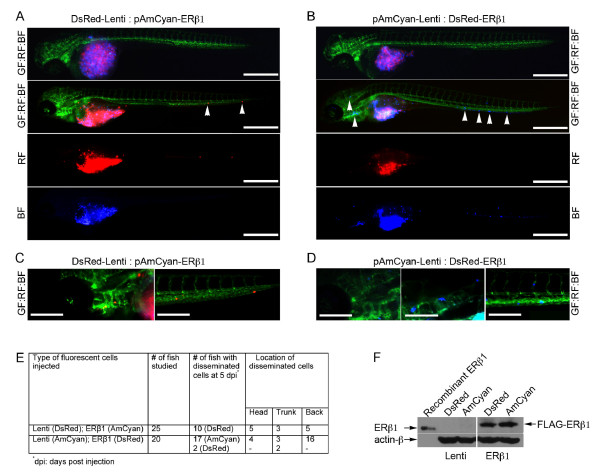
**ERβ1 inhibits MDA-MB-231 tumor cell invasion, dissemination and micrometastasis *in vivo***. Control (Lenti) and ERβ1-expressing MDA-MB-231 cells were stably transfected with pAmCyan or pCMCV-DsRed vector. A tumor cell suspension containing equal numbers of either DsRed-Lenti:AmCyan-ERβ1 cells **(A) **or AmCyan-Lenti:DsRed-ERβ1 cells **(B) **were injected into perivitelline space of 48 hpf embryos and tumor cell invasion and dissemination were detected using fluorescent microscopy at 5 dpi. The upper panels show the zebrafish 3 hpi. Arrowheads indicate disseminated tumor cells (Scale bar, 500 μm). (**C **and **D**) High magnification micrographs of A and B, respectively (scale bar, 100 μm). **(E) **Table showing the number of zebrafish injected with either DsRed-Lenti:AmCyan-ERβ1 or AmCyan-Lenti:DsRed-ERβ1 MDA-MB-231 cells, the number of zebrafish with disseminated human tumor cells and the number of the zebrafish with disseminated cells in different regions of the body. **(F) **DsRed-Lenti, AmCyan-Lenti, DsRed-ERβ1 and AmCyan-ERβ1 MDA-MB-231 cells were analyzed for ERβ1 expression by immunoblotting. (BF, blue filter; RF, red filter; GF, green filter).

To study the role of ERβ1 in regulating early events of the metastatic cascade, we used a zebrafish tumor model in which the *Tg(flk1:EGFP)*/casper zebrafish embryos were implanted with the highly metastatic human MDA-MB-231 cells. The *Tg(flk1:EGFP)*/casper embryos lack pigmentation and express green fluorescent protein (GFP) in the vascular system for direct visualization of vascular development [41]. Both control (Lenti) and ERβ1-expressing MDA-MB-231 cells were stably transfected with either DsRed or AmCyan fluorescent proteins. A mixture of either control-DsRed and ERβ1-AmCyan cells or control-AmCyan and ERβ1-DsRed cells were injected into the perivitelline cavity at 48 hours post-fertilization (hpf), at which time the immune system of the fish is not yet developed. The zebrafish were first imaged 3 h after implantation (Figure 5A, B, upper panels). Invasion and dissemination of DsRed and AmCyan cells were monitored daily in zebrafish. At five days post-injection (dpi), both DsRed and AmCyan MDA-MB-231 control cells had significantly disseminated away from the primary injection site, including the head and the tail regions, whereas ERβ1-expressing MD-MB-231 cells labeled with either DsRed or AmCyan remained at the primary site (Figure 5A-D). Out of 45 embryos that were injected with both control and ERβ1-expressing cells, 27 embryos had disseminated control cells, and only 2 embryos had disseminated control and ERβ1-expressing cells. However, in these two zebrafish, the ratio of control:ERβ1 disseminated cells was more than 8:1 (Figure 5E; Additional file 11, Figure S7). Our results show that the difference in metastatic potential between the control and the ERβ1-expressing cells is due to their different capacity to invade and disseminate.

### ERβ1 and E-cadherin levels are positively correlated in breast cancers

**Figure 6:**
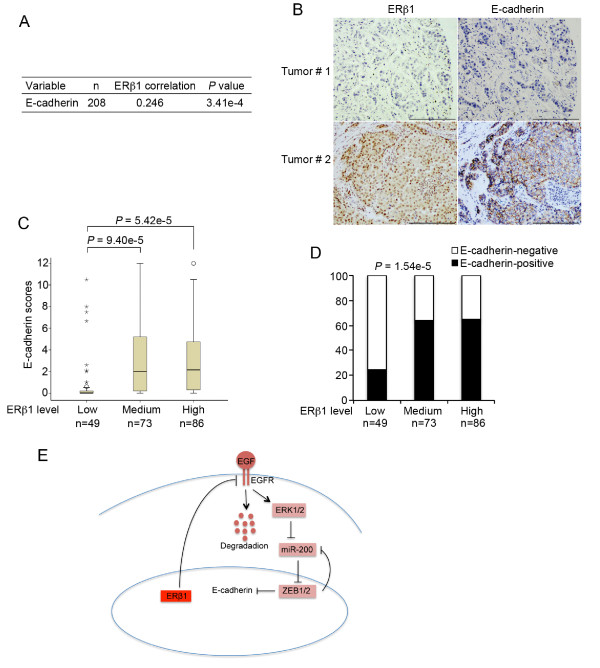
**ERβ1 levels positively correlate with E-cadherin in breast cancers**. **(A) **Pearson's correlation of ERβ1 expression with expression of E-cadherin. N equals the number of patients for whom data were available. **(B) **Representative images of ERβ1 and E-cadherin expression in two serial sections of the same tumor from two cases. Scale bars represent 200 μM. **(C) **ERβ1 and E-cadherin were box-plotted in the 208 breast cancer patients. The patients were divided into three groups based on ERβ1 expression scores in the tumors, representing low, medium and high expression of ERβ1. Any outliers were marked with a circle and extreme cases with an asterisk. Data were analyzed using one-way ANOVA test with Games-Howell's correction. **(D) **The percentage of E-cadherin-positive tumors was analyzed in the three groups of patients as described in C. Data were analyzed using Pearson's χ^2 ^test. **(E) **Proposed mechanism for how ERβ1 regulates EMT and influences invasion in breast cancer. EGFR promotes EMT in basal cells by activating ERK1/2, which in turn, by inducing the expression of ZEB1/2, results in the down-regulation of E-cadherin. This process requires repression of the expression of members of miR-200 family. By inducing the degradation of EGFR, ERβ1 sustains ERK1/2 inactive, up-regulates miR200a-b and miR-429, down-regulates ZEB1/2 and induces the expression of E-cadherin.

Since ERβ1 induces the expression of E-cadherin in breast cancer cells, we next examined the correlation of ERβ1 and E-cadherin protein levels in breast tumor samples. We utilized a tissue microarray of 240 primary untreated and unselected breast cancers. Clinical history and tumor characteristics (tumor type, age, size, grade, lymph node status, ERα, PR and HER2 status) that were available for 238 cases are summarized in Additional file 3, Table S2. ERβ1 and E-cadherin protein levels were determined by IHC using an ERβ1 specific antibody (PPG5/10; DAKO). The specificity of the ERβ1 antibody was confirmed by immunocytochemistry (Additional file 12 Figure S8A, B). A total of 32 samples were excluded from the analysis due to the absence of tumor or presence of benign tumor in the core. We carried out Pearson's correlation analysis with the information of ERβ1 and E-cadherin expression from the 208 cancers. Pearson's correlation analysis showed a strong positive correlation between ERβ1 and E-cadherin expressions (*P *= 3.41e-4) (Figure 6A).

To better understand the correlation between ERβ1 and E-cadherin, we divided the breast cancer samples into three groups based on ERβ1 levels defined by their expression scores and studied the difference of E-cadherin expression among the three groups. The expression of E-cadherin in each group was represented by its median score (Figure 6C) or positive percentage (Figure 6D). As shown in Figure 6C, the median scores of E-cadherin in tumors expressing low levels of ERβ1 were significantly smaller than in those with higher ERβ1 levels. Similarly, the positive percentage analysis for E-cadherin showed a positive correlation with ERβ1 levels (Figure 6D). These results are consistent with our findings that ERβ1 up-regulates E-cadherin in breast cancer cell lines.

## Discussion

Although basal-like breast cancers in general are associated with relatively poor prognosis, they are heterogeneous, including diverse subgroups in terms of chemotherapy response and risk of developing distant metastases [2,6,7]. Interestingly, ERβ1 positivity has recently been associated with better survival in triple-negative cancers that were treated with tamoxifen and inversely correlated with the expression of EGFR, an important marker in the immunohistochemical identification of basal-like cancers [24,25,36].

One process that has been attributed to primary tumor metastasis is EMT. Here we examined whether ERβ1 through regulating EMT can influence invasion and metastasis in basal-like cancers. ERβ1 repressed the mesenchymal spindle-shaped morphology of the MDA-MB-231 and Hs578T cells and enhanced cell-cell contact. ERβ1 altered the morphology of these cells in the absence of ligand. This is in agreement with our previous data showing increased transcriptional activity following expression of ERβ1 in MDA-MB-231 cells in the absence of ERβ agonists. The increased transcriptional activity in the absence of ligand was correlated with the phosphorylation of ERβ1 at ser-87 [28]. As a result of the changes in the morphology, ERβ1 inhibited migration and reduced the invasiveness of MDA-MB-231 cells. When control and ERβ1-expressing cells were injected into zebrafish embryos, only the control cells disseminated to distant sites suggesting that ERβ1 functions as a crucial anti-invasive factor. Given that expression of EMT markers and cadherin switching have been reported to correlate with the basal-like phenotypes in *in vitro *model systems and in specimens from patients [7], we examined whether ERβ1 inhibits invasion and migration by regulating EMT in cells with basal characteristics. ERβ1 was found to induce the expression of E-cadherin by inhibiting its transcriptional repressors ZEB1/2 and up-regulating the miR-200a, miR-200b and miR-429, which correlate with the epithelial breast cancer phenotype (Figure 6E).

ERK2 has recently been shown to affect the ZEB1/2 regulatory pathway of E-cadherin expression in human mammary cells [17,18]. ERK1/2 are activated by diverse pathways including that initiated by EGFR [38]. Overexpression of EGFR promotes migration and invasion of basal cells and its expression correlates with poor survival in basal-like cancers [36,37]. Since ERβ1 was found to inhibit EMT by down-regulating the ZEB1/2 pathway in basal-like cells, we tested whether repression of EGFR and ERK1/2 signaling are involved in ERβ1-mediated up-regulation of E-cadherin and the subsequent inhibition of cell migration and invasion. Indeed, ERβ1 induced a decrease in EGFR protein levels without altering the transcription of the *EGFR *gene followed by down-regulation of the phosphorylated ERK1/2 forms. Induction of EGFR signaling in ERβ1-expressing cells through up-regulation of EGFR or treatment of the cells with EGF reversed the ERβ1-dependent epithelial phenotype, suggesting that EGFR is a critical factor in the ERβ1-mediated regulation of EMT.

Given that inhibition of transcription was not involved in ERβ1-mediated down-regulation of EGFR, we examined whether ERβ1 promotes degradation of the tyrosine kinase receptor. EGFR degradation is a complex process that involves ubiquitylation of the activated receptor by the E3 enzyme Cbl and subsequent proteolysis by proteosomal and lysosomal hydrolases [39]. ERβ1 was found to induce ubiquitylation and degradation of EGFR by enhancing the EGFR-c-Cbl association. Ubiquitylation is an important process of a negative regulatory circuit that terminates EGFR signaling by targeting the receptor for degradation [42]. Our data show for first time that ERβ1, by inducing these negative feedback pathways, is likely to exert a role of EGFR inhibitor and tumor suppressor function.

Interestingly, it has recently been shown that ERβ decreases the expression of insulin-like growth factor II-mRNA binding protein 3 (IMP-3) by repressing EGFR transcription in MDA-MB-231 cells [43]. In our study, the transcription of EGFR was not altered when ERβ1 was expressed or knocked down in MDA-MB-231 and Hs578T basal-like cells. Instead, as mentioned above, ERβ1 promotes degradation of EGFR by inducing its ubiquitylation in both MDA-MB-231 and Hs578T cells.

By examining 208 clinical breast cancer specimens, we found that the expression of ERβ1 was significantly associated with the expression of E-cadherin. This correlation has not previously been reported. However, since the discovery of ERβ, it has been shown that the association of ERβ to other clinicopathological indicators is likely to be divergent in different breast cancer cohorts analyzed by IHC using different ERβ antibodies. The tumor cohort examined in our study included a different number of HER2-positive (14.5%) and probably triple-negative breast cancers compared with the cohorts utilized in some of the recent studies that examined large number of samples with well-validated antibodies (HER2-positive, Honma *et al*. 2008: 5.25% and Novelli *et al*. 2008: 31.9%) [24,25,44,45]. Such differences in the characteristics of the clinical cancer samples as well as differences in the specificity of the ERβ antibodies used in these studies, may explain why the correlation between ERβ1 and E-cadherin expression has not been previously observed. This positive ERβ1-E-cadherin association is consistent with the ERβ1-mediated up-regulation of E-cadherin observed in breast cancer cells. It is possible that there are some limitations in the relevance of these results since the level of ERβ1-expression achieved in our cells may not reflect the levels of expression seen in clinical samples. Despite these limitations, taken together, our results propose a role for ERβ1 in up-regulating E-cadherin in breast cancer cells. This suggests that the low ERβ1 levels may be the primary cause of low E-cadherin expression and induction of EMT in some breast cancers. Since EMT correlates with a group of basal-like breast cancers that often develop metastases in distant sites [7], ERβ1 may play a crucial role in repressing invasive behavior and inhibiting metastasis in this subset of breast cancers. Our data show that ERβ1 impedes EMT and influences invasion by down-regulating EGFR, which is expressed in basal-like cancers. These results strengthen the possibility that ERβ1 can help to identify patients with basal-like cancer with lower risk to develop metastasis.

## Conclusions

Basal-like breast cancers that show unfavorable prognosis and often develop distant metastases are associated with EMT. Our findings indicate that ERβ1 inhibits EMT and reduces the invasiveness of basal-like breast cancer cells by up-regulating the epithelial marker E-cadherin. ERβ1 induces the expression of E-cadherin by down-regulating EGFR, an oncogenic factor that is expressed in basal-like cancers. ERβ1 was found to terminate EGFR signaling by targeting the receptor for degradation. Our data support the notion that ERβ1 can serve as a clinical marker to identify patients with basal-like cancer that have lower risk to develop metastasis.

## Abbreviations

BSA: bovine serum albumin; CK: cytokeratin; DCC: dextran-coated charcoal; DCIS: ductal carcinoma *in situ*; dpi: days post-injection; EGFR: epidermal growth factor receptor; EMT: epithelial to mesenchymal transition; ERα: estrogen receptor α; ERβ: estrogen receptor β; ERKs: extracellular-signal-regulated kinases; FCS: fetal calf serum; GFP: green fluorescent protein; hpf: hours post-fertilization; IACUC: Institutional Animal Care and Use Committee; IHC: immunohistochemistry; IMP-3: insulin-like growth factor II mRNA binding protein 3; PBS: phosphate-buffered saline; RT: room temperature; RT-PCR: reverse transcription polymerase chain reaction; TGF-β: transforming growth factor β

## Competing interests

The authors declare that they have no competing interests.

## Authors' contributions

CT conceived, designed and supervised the study, performed or participated in all experiments, their analysis and interpretation, and wrote the manuscript. JAG supervised research and edited the manuscript. GR participated in the *in vitro *experiments with the cell lines and in the immunohistochemical staining of the tissue sections. FN participated in most of the cell-based studies. RH and CWM performed the experiments with the transplantation of human cells in zebrafish. MB supervised the zebrafish xenotransplantation assay and participated in editing the manuscript. AK assisted in the experiments with the microRNAs. PQ assisted in the clinical data analysis. SK participated in the design of the clinical study and evaluated the immunohistochemical staining. FJE and AT participated in the design of the clinical study and supervised the analysis of the clinical breast cancer specimens. All authors read and approved the final manuscript.

## Supplementary Material

Additional file 1 **Table 1S. Oligonucleotides used in qPCR**. The table lists the sequences of the oligonucleotides used in qPCR. Click here for file

Additional file 2 **Supplementary materials and methods**. The file contains supplementary information for the zebrafish lines used in xenotransplantation study. Click here for file

Additional file 3 **Table S2. Clinicopathological characteristics of 238 breast cancer patients**. The table contains the clinicopathological characteristics of 238 breast cancer patients. Click here for file

Additional file 4 **Figure S1. Functional analysis of ERα and ERβ1 in MDA-MB-231 cells**. The figure shows the functionality of ERα and ERβ1 in MDA-MB-231 cells. Click here for file

Additional file 5 **Supplementary figure legends**. The file contains the figure legends for the supplementary figures S1-S8. Click here for file

Additional file 6 **Figure S2. Regulation of EMT markers by ERβ1**. Description: The figure shows how ERβ1 regulates some of the EMT markers. Click here for file

Additional file 7 **Figure S. ERβ1 does not alter the intracellular localization of SNAIL**. The figure shows how ERβ1 affects the intracellular localization of SNAIL. Click here for file

Additional file 8 **Figure S4. ERβ1 regulates the expression of miR-200a, miR-200b and miR-429**. The figure shows the regulation of miR-200a, miR-200b and miR-429 by ERβ1 in Hs578T cells. Click here for file

Additional file 9 **Figure S5. Regulation of miR-200c, miR-141 and miR-205 by ERβ1**. The figure shows the regulation of miR-200c, miR-141 and miR-205 by ERβ1. Click here for file

Additional file 10 **Figure S6. Differences in the expression of EGFR between the ERα-positive **(MCF-7) and the triple-negative (MDA-MB-231 and Hs578T) cells. The figure shows the different expression levels of EGFR in MCF-7, MDA-MB-231 and Hs578T breast cancer cells. Click here for file

Additional file 11 **Figure S7. Dissemination patterns of ERβ1-expressing cells in zebrafish**. The figure shows the dissemination patterns of ERβ1-expressing cells in zebrafish. Click here for file

Additional file 12 **Figure S8. Validation of the anti-ERβ1 antibody by immunocytochemistry**. The figure shows the specificity of the anti-ERβ1 antibody used in immunohistochemistry. Click here for file
