# Supplementary material for: ERβ1 represses basal-like breast cancer epithelial to mesenchymal transition by destabilizing EGFR
Source: Breast Cancer Res. 2012 Nov 16;14(6):R148. doi: 10.1186/bcr3358 (PMC4053135; doi:10.1186/bcr3358)
Supplement: Additional file 1 — Table 1S. Oligonucleotides used in qPCR. The table lists the sequences of the oligonucleotides used in qPCR. [file bcr3358-S1.PDF]

Table S1

Oligonucleotides used in qPCR

ERβ1 for	GCT CAA TTC CAG TAT GTA CC
ERβ1 rev	GGA CCA CAT TTT TGC ACT
E-cadherin for	CCC ACC ACG TAC AAG GGT C
E-cadherin rev	CTG GGG TAT TGG GGG CAT C
EGFR-Isoform1 for #1	CGA GAC CCC CAG CGC TAC CT
EGFR-Isoform1 rev#1	CGG CAT CCA CCA CGT CGT CC
EGFR-Isoform1 for #2	GCCCCCACTGCGTCAAGACC
EGFR-Isoform1 rev #2	ACCTGGCCCAGTGCATCCGT
Vimentin for	CGA AAA CAC CCT GCA ATC TT
Vimentin rev	CTG GAT TTC CTC TTC GTG GA
Twist for	TGC ATG CAT TCT CAA GAG GTT GCA
Twist rev	CTA TGG TTT TGC AGG CCA GT
Snail for	CTC TAG GCC CTG GCT GCT AC
Snail rev	TCTGAGTGGGTCTGGAGGTG
miR 200b	GGT AAT ACT GCC TGG TAA TGA TGA
miR-141	GCT AAC ACT GTC TGG TAA AGA TGG
miR-200a	CGT AAC ACT GTC TGG TAA CGA TGT
miR-200c	CTG CCG GGT AAT GAT GGA
miR-429	CGC TAA TAC TGT CTG GTA AAA CCG T
