# Supplementary material for: ERβ1 represses basal-like breast cancer epithelial to mesenchymal transition by destabilizing EGFR
Source: Breast Cancer Res. 2012 Nov 16;14(6):R148. doi: 10.1186/bcr3358 (PMC4053135; doi:10.1186/bcr3358)
Supplement: Additional file 2 — Supplementary materials and methods. The file contains supplementary information for the zebrafish lines used in xenotransplantation study. [file bcr3358-S2.PDF]

Supplementary Materials and Methods

Zebrafish lines used in xenotransplantation study

Zebrafish lines used in these studies included the transgenic strain expressing enhanced green fluorescent protein (EGFP) under the Flk1 promoter, *Tg(Flk-1;EGFP)*, which was a gift from Dr. Daniel Wagner (Rice University) and allows for visualization of the vascular system. In addition, the pigmentation mutant *casper* line that demonstrates a complete lack of all melanocytes and iridophores in both embryogenesis and adulthood was purchased from Zebrafish International Resource Center (ZIRC). This fish is almost entirely transparent. The casper *Tg(Flk-1;EGFP)* zebrafish was created by crossing *Tg(Flk-1;EGFP)* with the casper line.
