# Supplementary material for: ERβ1 represses basal-like breast cancer epithelial to mesenchymal transition by destabilizing EGFR
Source: Breast Cancer Res. 2012 Nov 16;14(6):R148. doi: 10.1186/bcr3358 (PMC4053135; doi:10.1186/bcr3358)
Supplement: Additional file 3 — Table S2. Clinicopathological characteristics of 238 breast cancer patients. The table contains the clinicopathological characteristics of 238 breast cancer patients. [file bcr3358-S3.PDF]

Table S2

Clinicopathological characteristics of 238 breast cancer patients

Characteristic	No. of patients	%
Age at diagnosis, years		
>50	182	76.5
≤50	56	23.5
Tumor size, cm		
0.1-1	12	5
1-2	77	32.3
2-5	131	55
>5	18	7.5
Invasive grade		
I	33	14.2
II	92	39.6
III	107	46.1
Not recorded	6	
Nodal status		
Node-positive	113	47.4
Node-negative	125	52.5
Tumor type		
Invasive ductal carcinoma	192	80.6
Invasive lobular carcinoma	14	5.8
Tubular carcinoma	5	2.1
Mucinous carcinoma	5	2.1
Medullary carcinoma	2	0.8
Other rare types and mixed	20	8.4
ERα status		
Positive	178	74.7
Negative	60	25.2
PR status		
Positive	128	53.7
Negative	110	46.2
HER2 status		
Positive	33	14.5
Negative	194	85.4
Not identified	11	
Treatment		
Adjuvant Endocrine	186	78
Adjuvant Chemotherapy	69	28.9
Adjuvant Radiotherapy	139	58.4
