# Supplementary figures and images for: ERβ1 represses basal-like breast cancer epithelial to mesenchymal transition by destabilizing EGFR
Source: Breast Cancer Res. 2012 Nov 16;14(6):R148. doi: 10.1186/bcr3358 (PMC4053135; doi:10.1186/bcr3358)

Figure S1

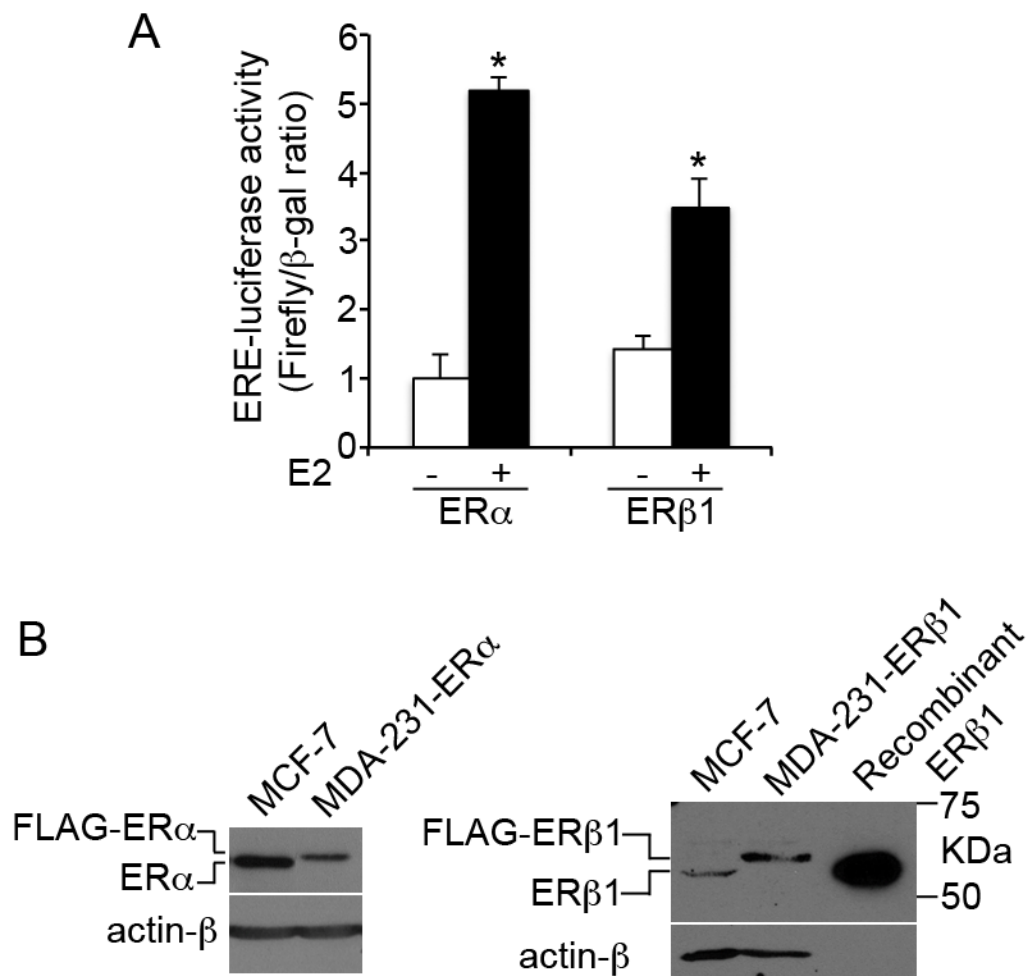

Supplement: Additional file 4 — Figure S1. Functional analysis of ERα and ERβ1 in MDA-MB-231 cells. The figure shows the functionality of ERα and ERβ1 in MDA-MB-231 cells. [file bcr3358-S4.PDF]

Figure S2

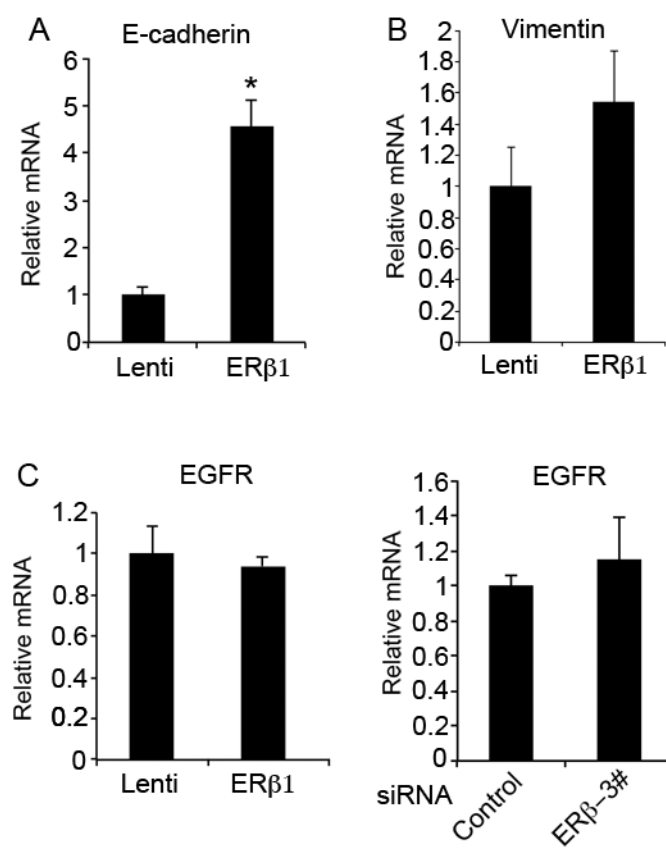

Supplement: Additional file 6 — Figure S2. Regulation of EMT markers by ERβ1. Description: The figure shows how ERβ1 regulates some of the EMT markers. [file bcr3358-S6.PDF]

Figure S3

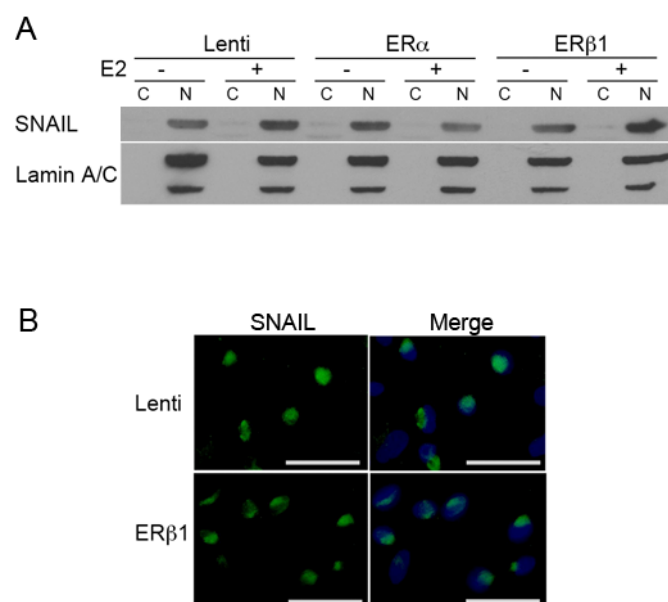

Supplement: Additional file 7 — Figure S. ERβ1 does not alter the intracellular localization of SNAIL. The figure shows how ERβ1 affects the intracellular localization of SNAIL. [file bcr3358-S7.PDF]

Figure S4

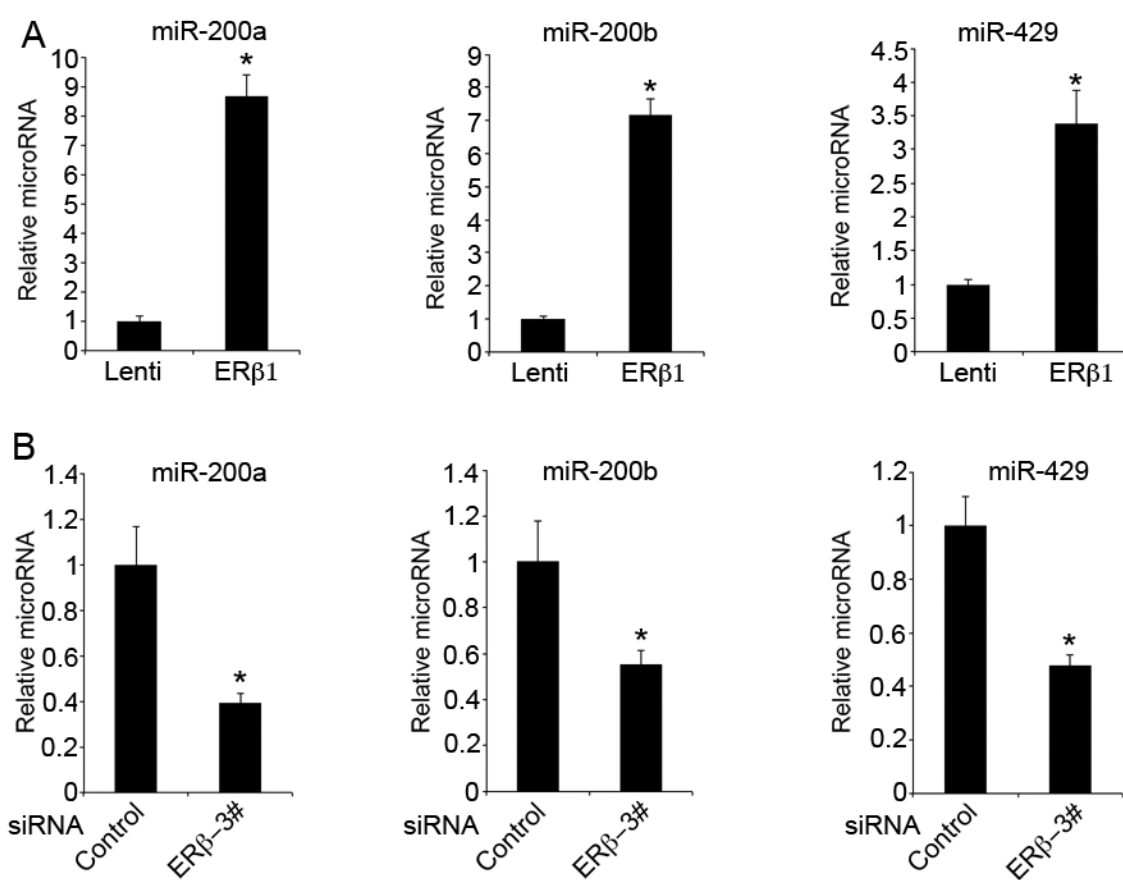

Supplement: Additional file 8 — Figure S4. ERβ1 regulates the expression of miR-200a, miR-200b and miR-429. The figure shows the regulation of miR-200a, miR-200b and miR-429 by ERβ1 in Hs578T cells. [file bcr3358-S8.PDF]

Figure S5

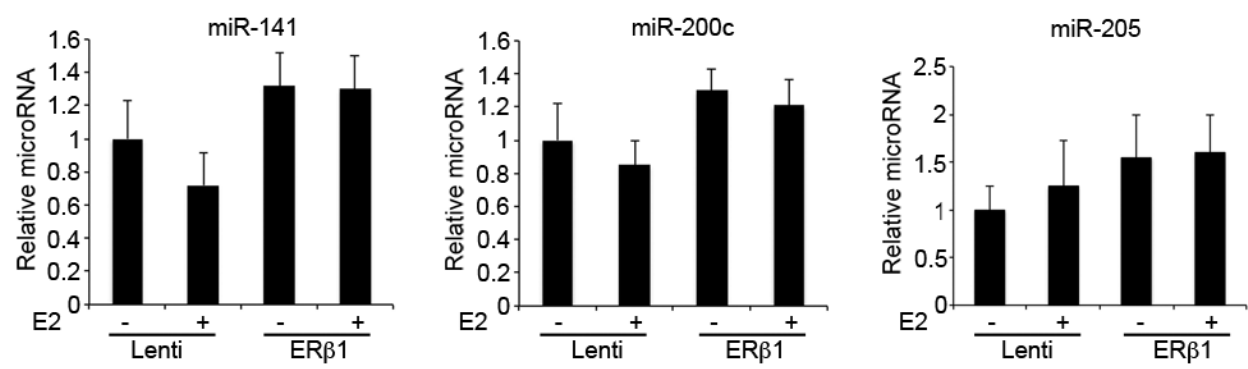

Supplement: Additional file 9 — Figure S5. Regulation of miR-200c, miR-141 and miR-205 by ERβ1. The figure shows the regulation of miR-200c, miR-141 and miR-205 by ERβ1. [file bcr3358-S9.PDF]

Figure S6

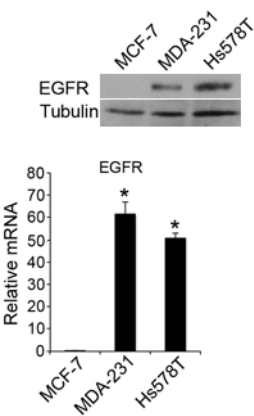

Supplement: Additional file 10 — Figure S6. Differences in the expression of EGFR between the ERα-positive (MCF-7) and the triple-negative (MDA-MB-231 and Hs578T) cells. The figure shows the different expression levels of EGFR in MCF-7, MDA-MB-231 and Hs578T breast cancer cells. [file bcr3358-S10.PDF]

Figure S7

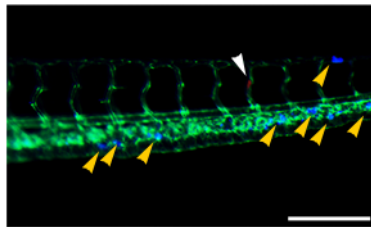

Supplement: Additional file 11 — Figure S7. Dissemination patterns of ERβ1-expressing cells in zebrafish. The figure shows the dissemination patterns of ERβ1-expressing cells in zebrafish. [file bcr3358-S11.PDF]

Figure S8

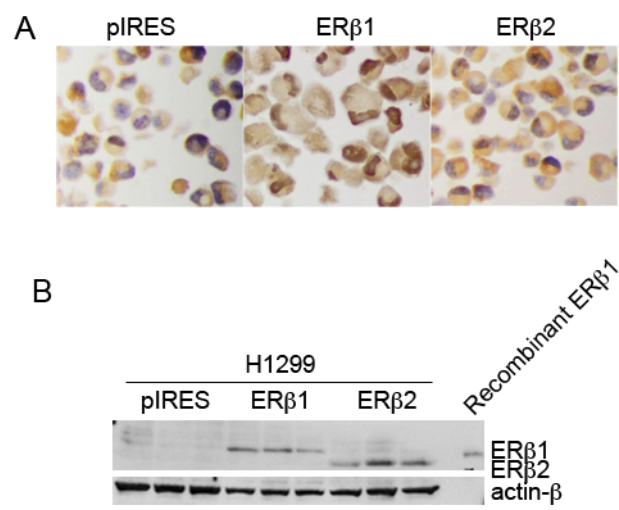

Supplement: Additional file 12 — Figure S8. Validation of the anti-ERβ1 antibody by immunocytochemistry. The figure shows the specificity of the anti-ERβ1 antibody used in immunohistochemistry. [file bcr3358-S12.PDF]
