# Supplementary material for: ERβ1 represses basal-like breast cancer epithelial to mesenchymal transition by destabilizing EGFR
Source: Breast Cancer Res. 2012 Nov 16;14(6):R148. doi: 10.1186/bcr3358 (PMC4053135; doi:10.1186/bcr3358)
Supplement: Additional file 5 — Supplementary figure legends. The file contains the figure legends for the supplementary figures S1-S8. [file bcr3358-S5.PDF]

Supplementary Figure Legends

Figure S1. Functional analysis of ERα and ERβ1 in MDA-MB-231 cells

(A) Luciferase reporter assay in ERα and ERβ1-expressing MDA-MB-231 cells demonstrating similar activation of an ERE-luciferase reporter following incubation with 10 nM E2. The data in the graph represent the mean of three separate experiments, SEM and *P<0.05 are indicated. **(B)** Immunoblots of ERα and ERβ1 in lysates from parental MCF-7 cells and ERα- and ERβ1-expressing MDA-MB-231 cells. MCF-7 cells express relatively high levels of ERα and relatively low levels of ERβ1. The immunoblots indicate lower expression of ERα in ERα-expressing MDA-MB-231 cells compared to the endogenous ERα in MCF-7 cells and higher expression of ERβ1 in ERβ1-expressing MDA-MB-231 cells compared to endogenous ERβ1 in MCF-7 cells.

Figure S2. Regulation of EMT markers by ERβ1

(A) Control (Lenti) and ERβ1-expressing (ERβ1) Hs578T cells were analyzed for E-cadherin expression by qPCR. **(B)** Control and ERβ1-expressing MDA-MB-231 cells were analyzed for Vimentin expression by qPCR. **(C)** Left: control and ERβ1-expressing MDA-MB-231 cells were analyzed for EGFR expression by TaqMan mRNA assay. Right: MDA-MB-231 cells were transiently transfected with control or ERβ siRNA (3#) and analyzed for EGFR expression by TaqMan mRNA assay. The values in the graphs represent the mean of three separate experiments with SEM and p value (*) ≤ 0.05% indicated.

Figure S3. ERβ1 does not alter the intracellular localization of SNAIL

(A) Cytoplasmic (c) and nuclear (n) extracts were prepared from EtOH- and E2-treated control (Lenti), ERα- and ERβ1-expressing MDA-MB-231 cells and analyzed for SNAIL expression by immunoblotting. **(B)** SNAIL was visualized by immunofluorescence in control (Lenti) and ERβ1-expressing (ERβ1) MDA-MB-231 cells. In the pictures, the merge images were obtained from SNAIL staining (green: FITC) and nuclear staining (blue: DAPI). Scale bars represent 20 µm.

Figure S4. ERβ1 regulates the expression of miR-200a, miR-200b and miR-429

(A) Control (Lenti) and ERβ1-expressing Hs578T cells were analyzed for miR-200a-b and miR-429 expression by qPCR. **(B)** Hs578T cells were transiently transfected with control or ERβ siRNA (3#) and analyzed for miR-200a-b and miR-429 expression by qPCR. The data in the graphs represent the mean of three separate experiments (±SEM) with *p* value (*) ≤ 0.05%.

Figure S5. Regulation of miR-200c, miR-141 and miR-205 by ERβ1

Control (Lenti) and ERβ1-expressing MDA-MB-231 cells were incubated in 5% DCC-FCS media in the absence or presence of E2 for 24 h and analyzed for the expression of miR-200c, miR-141 and miR-205 by qPCR. The data in the graphs represent the mean of three separate experiments with SEM.

Figure S6. **Differences in the expression of EGFR between the ERα-positive (MCF-7) and the triple-negative (MDA-MB-231 and Hs578T) cells**

MCF-7, MDA-MB-231 and Hs578T cells were analyzed for EGFR expression by immunoblotting and TaqMan mRNA assay. The values represent the fold change compared with the MCF-7 cells, 3 independent experiments performed in triplicate, SEM and *P<0.05 are indicated.

Figure S7. Dissemination patterns of ERβ1-expressing cells in zebrafish

A cell suspension containing equal numbers of AmCyan-Lenti and DsRed-ERβ1 MDA-MB-231 cells were injected into perivitelline space of 48 h post-fertilization embryos and tumor cell invasion, dissemination and mictometastasis were detected using fluorescent microscopy at 5 days post-injection. The white arrowhead indicates the DsRed-ERβ1 disseminated cell, while yellow arrowheads show the AmCyan-Lenti disseminated cells. Note that this represents one of the two zebrafish in which ERβ1-expressing disseminated cells were detected. However, the ratio or control:ERβ1-expressing disseminated cells is high (only one ERβ1-expressing cell in 8 control cells). Scale bar represents 100 µm.

Figure S8. Validation of the anti-ERβ1 antibody by immunocytochemistry

(A) Formalin-fixed control (pIRES), ERβ1- and ERβ2-expressing H1299 lung cancer cells were embedded in paraffin, the cell blocks were cut at 5μm intervals and used for IHC staining with the same anti-ERβ1 antibody (PPG5/10, Dako) used in IHC of the clinical breast cancer samples. The left panel represents control cells that are ERβ1-negative, the middle panel shows the ERβ1-expressing cells and the right panel indicates the ERβ2-expressing cells that are

ERβ1-negative. **(B)** Immunoblotting of ERβ in lysates from control, ERβ1- and ERβ2-expressing H1299 lung cancer cells using the 14C8 anti-ERβ antibody (Genetex) that detects both ERβ1 and ERβ2.
